# Fabrication of Structured Surface Functional Layers for Enhanced Performance of Ag_2_Se-Based Photothermoelectric Detectors

**DOI:** 10.3390/mi17060739

**Published:** 2026-06-18

**Authors:** Gailing Tian, Rui Guo, Yun Gong, Wenjing Zhang, Weipeng Shi, Yi Chen, Yonghua Wang, Jinglong Wen, Dan Liu, Chenyang Xue

**Affiliations:** State Key Laboratory of Extreme Environment Optoelectronic Dynamic Measurement Technology and Instrument, North University of China, Taiyuan 030051, China; 18803510309@139.com (G.T.); 18534715853@139.com (R.G.); 15287461467@163.com (Y.G.); zb2006040103@163.com (W.Z.); swpjjt@163.com (W.S.); chenyi@nuc.edu.cn (Y.C.); wangyonghua@nuc.edu.cn (Y.W.); 20200002@nuc.edu.cn (J.W.); xuechenyang@nuc.edu.cn (C.X.)

**Keywords:** Ag_2_Se, surface microstructure, photothermoelectric detectors

## Abstract

To address the issues of low light absorption efficiency and limited temperature gradient distribution in conventional planar Ag_2_Se-based photothermoelectric (PTE) detectors, this paper proposes a structured design strategy for the surface functional layer. Ag_2_Se-based PTE detectors with periodic surface microstructure arrays were fabricated using photolithography, and the influence of surface structure on the device’s PTE response performance was systematically investigated. The results indicate that surface microstructures can enhance light absorption and localized photothermal conversion efficiency, thereby increasing the PTE output voltage. However, they also lengthen the thermal diffusion path and reduce the dynamic response speed. When the structural pitch is 6.7 um, the device exhibits optimal overall detection performance within the measured spectral range of 405–950 nm. Under irradiation at a wavelength of 950 nm and a laser power density of 120 mW/cm^2^, the device achieved a voltage sensitivity of 0.14 mV/W. This study reveals the trade-off between enhancing the response performance and response speed of Ag_2_Se-based PTE detectors through surface structural design, providing experimental evidence and design guidance for rationally optimizing device structural parameters and realizing room-temperature PTE detection.

## 1. Introduction

With the rapid development of information technology and intelligent sensing systems, there is an increasingly urgent need for novel photodetectors that combine high sensitivity, fast response, self-powered operation [1], and broadband response [2,3]. Traditional photodetectors are primarily based on the mechanism of photo-generated carrier separation [4], their performance is often limited by the bandgap width and carrier mobility of the semiconductor material, and they typically require an external bias for operation [5]. In contrast, photothermoelectric (PTE) detectors rely on the cascaded effects of photothermal and thermoelectric conversion [6]. They offer advantages such as no need for bias voltage [7], operation at room temperature, and a response wavelength determined by the absorbing material, demonstrating broad application prospects in fields such as remote sensing and imaging [8,9,10].

Among numerous thermoelectric materials, Ag_2_Se has emerged as an ideal candidate for high-performance PTE detectors due to its excellent thermoelectric properties and good environmental stability [11,12]. However, traditional Ag_2_Se-based PTE detectors with planar structures typically suffer from limited light absorption efficiency and uneven temperature gradient distribution, which hinder further improvements in their responsivity and detection efficiency. To address these bottlenecks, researchers have increasingly focused on the surface structuring of the photosensitive layer in recent years [13,14,15]. By introducing micro–nanoscale periodic or non-periodic structural arrays (such as gratings, pores, pillars, and pyramids) onto the device surface [16], the interaction between light and the material can be significantly altered. Specifically, these surface structures can induce multiple light scattering, diffraction coupling, and surface plasmon effects, thereby effectively extending the light propagation path within the absorption layer, enhancing the effective optical path length, and increasing the light absorption density per unit volume. More importantly, surface structures can cause a redistribution of local optical field energy, forming hot spot regions that significantly enhance localized photothermal conversion efficiency and create high-intensity [17], non-uniform temperature gradient fields along the in-plane direction of the thin film. This temperature gradient can directly drive more efficient thermoelectric conversion processes, thereby substantially improving the device’s responsivity and detection sensitivity without altering the material’s thermoelectric figure of merit (ZT) [18].

Recent studies on self-powered and broadband photodetectors have demonstrated that heterojunction engineering, interface modulation, pyro-phototronic enhancement, and PTE conversion can extend operating wavelength ranges and improve device sensitivity [2,4,7,9,15]. Representative examples include La-doped PbSe-film and WSe_2_/Si heterojunction detectors with broadband responses extending to 1550 nm [2,4], all-inorganic perovskite tandem photodetectors enhanced by pyro-phototronic effects [9], PtTe_2_/black-silicon detectors optimized for 1064 nm near-infrared response [15], and Ag-decorated carbon-nanotube films that use the PTE effect for infrared detection [7]. These reports show that high-performance photodetection generally requires not only strong light absorption and efficient carrier or thermal-energy conversion but also calibrated noise analysis for evaluating the true detection limit. In this context, surface-structure engineering is a practical route for improving Ag_2_Se-based PTE detectors while preserving room-temperature and zero-bias operation.

This work proposes a novel strategy based on the structured design of Ag_2_Se surface functional layers. Using standard photolithography processes, a functional layer featuring a periodic structural array was fabricated on the surface of Ag_2_Se-based PTE detectors. By systematically controlling the geometric parameters of the surface structure, the study systematically investigated the influence of the surface functional layer structure on the device’s light absorption characteristics, photothermal conversion capability, and ultimate PTE response performance. This study not only reveals the crucial role of surface structural design in optimizing the performance of Ag_2_Se-based PTE detectors but also provides a viable path for the development of high-performance, integrable novel room-temperature PTE detectors.

## 2. Experimental Section

### 2.1. Preparation of PTE Detectors

**Figure 1:**
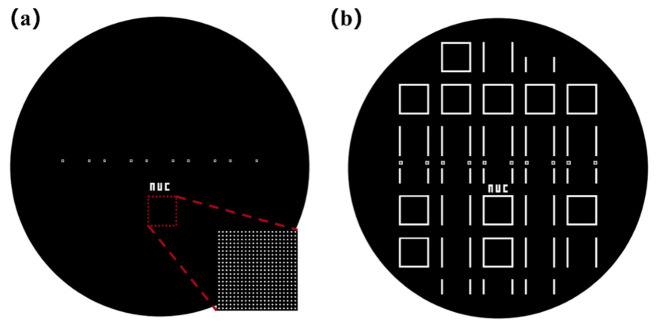
Mask pattern: (**a**) microstructure layer and magnified region, (**b**) electrode layer pattern.

Ag_2_Se-based PTE detectors with patterned surface functional layers were fabricated using MEMS photolithography and stripping techniques. The specific fabrication process includes magnetron sputtering, photolithography, and stripping, as detailed below. The photolithography process is a key technology in semiconductor manufacturing; it enables the precise definition of microstructures at the micrometer or nanometer scale and the transfer of these patterns onto the substrate [19]. The precision and resolution of the photolithography process directly affect the performance and integration level of the device. The patterning of the Ag_2_Se surface functional layer structure and electrodes is achieved through the photolithography process. Exposure and development are critical steps in photolithography, used to form the desired microscopic patterns on the photoresist [20]. In the photolithography process, a mask is a precision-manufactured plate with transparent and opaque regions, used to define the patterns of microcircuits or other structures during the process. The mask transfers the pattern onto a substrate coated with photoresist by blocking or allowing light to pass through its transparent sections. To fabricate Ag_2_Se-based PTE detectors with a structured surface functional layer, a two-layer mask design was employed, primarily created using L-edit v16.3 software. Based on their functions, the layers are primarily divided into a structural layer and an electrode layer. Since positive photoresist is used in this experiment, the masks are designed such that the patterned areas are exposed to light, while the non-patterned areas are shielded from light. Figure 1a shows mask M1, which corresponds to the Ag_2_Se structural layer; the pattern on this layer includes the micro–nanostructures on the device surface. Figure 1b shows mask M2, which corresponds to the Ag electrode layer.

The surface functional layer of the PTE detector was prepared using magnetron sputtering, a plasma-based physical vapor deposition technique that is simple and efficient. The resulting film is dense and uniform, and its composition and thickness can be precisely controlled. The sputtering parameters for the Ag_2_Se layer were a sputtering power of 50 W, a sputtering pressure of 1.5 Pa, and a sputtering time of 15 min; for the Ag_2_Se structural layer, the same sputtering power and pressure were used, with a sputtering time of 30 min.

**Figure 2:**
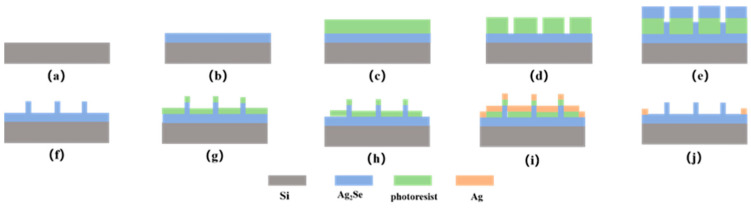
Process flow for fabricating Ag_2_Se-based detectors with structured surface functional layers: (**a**) substrate preparation, (**b**) magnetron sputtering of Ag_2_Se, (**c**) resist coating, (**d**) lithography patterning, (**e**) magnetron sputtering of Ag_2_Se, (**f**) lift-off, (**g**) resist coating, (**h**) lithography patterning, (**i**) magnetron sputtering of Ag electrodes, (**j**) lift-off.

The patterning of the Ag_2_Se surface functional layer structure was achieved using an exfoliation process. When combined with photolithography, this process constitutes a microfabrication technique for producing micro–nanoscale patterns. Its core advantage lies in fundamentally avoiding plasma damage or chemical corrosion that may occur during etching processes, thereby effectively protecting the sensitive substrate materials beneath. This method overcomes material limitations, offers high-resolution patterning potential, and simplifies the deposition process for multilayer films. The photoresist serves to form the pattern during the lift-off process. After coating, due to the step formed by the difference in thickness between the photoresist and the substrate, the film layer deposited on the photoresist becomes disconnected from the film layer deposited on the substrate. This allows the photoresist to react with the stripping solution, causing the film layer on the photoresist to peel off as the photoresist dissolves. Therefore, the thickness of the photoresist is a critical parameter; typically, the ratio of photoresist thickness to deposited film thickness is ≥3. In this study, to ensure successful photoresist stripping, the RDP-2100P photoresist (Nantong Jingai Microelectronics Technology Co., Ltd., Nantong, China) is considered for the photolithography and stripping process, and acetone (Nantong Jingai Microelectronics Technology Co., Ltd., Nantong, China) is selected as the stripping solution. Acetone is an organic solvent commonly used for cleaning and removing photoresist. It has strong solvent power and can dissolve many organic compounds. The fabrication process for Ag_2_Se-based detectors with a structured surface functional layer is shown in Figure 2.

The specific procedure is as follows: first, prepare and clean the silicon wafers. Select 4-inch single-polished silicon wafers and clean them thoroughly, as shown in Figure 2a. The cleaning procedure includes removing organic contaminants with an acetone solution, replacing and removing residual acetone and other organic solvents from the wafer surface with an ethanol (Tianjin Kaitong Chemical Reagent Co., Ltd., Tianjin, China) solution, rinsing thoroughly with deionized water (Shanghai Huazhen Technology Co., Ltd., Shanghai, China), and finally purging with a high-purity nitrogen gun until the wafer is completely dry. Second, magnetron sputtering of the Ag_2_Se functional layer. Use magnetron sputtering technology (CY-MSH500X-III-DCDCRF-SS, Zhengzhou Chengyue Scientific Instrument Co., Ltd., Zhengzhou, China) to deposit an Ag_2_Se thin film onto the silicon wafer, as shown in Figure 2b. Sputter Ag_2_Se using direct current (DC) at a sputtering power of 50 W, a sputtering pressure of 1.5 Pa, and a sputtering time of 15 min. The magnetron sputtering process involves bombarding a target material with high-energy particles within a vacuum chamber, causing atoms from the target to be sputtered onto the substrate surface to form a film. The thickness and deposition rate of each layer are precisely controlled to ensure interfacial quality and thickness uniformity. Third, pattern the surface functional layer structure. Photolithography was performed using the first mask, M1, to pattern the surface functional layer structure, as shown in Figure 2c,d. First, photoresist was uniformly coated onto the silicon wafer surface and pre-baked. Then, the structural pattern was exposed through mask M1, followed by development to remove the exposed photoresist. Fourth, prepare the surface functional layer structure. Ag_2_Se is deposited as the structural layer using magnetron sputtering, followed by a stripping process to remove excess photoresist with acetone, leaving Ag_2_Se in the patterned areas. As shown in Figure 2e,f, a surface functional layer structure with a specific pattern is formed. Fifth, pattern the electrode layer. A second mask, M2, is used for photolithography to pattern the electrode layer, as shown in Figure 2g,h. The photolithography steps are similar to the previous step: photoresist is uniformly coated onto the silicon wafer surface, followed by a pre-bake, and then aligned with the pattern of the structural layer for exposure. Sixth, there is electrode preparation. Ag is deposited as the electrode layer using magnetron sputtering. A stripping process is then performed, using acetone to remove excess photoresist and retain the Ag electrode in the patterned areas. As shown in Figure 2i,j, electrodes with a specific pattern are formed.

**Figure 3:**
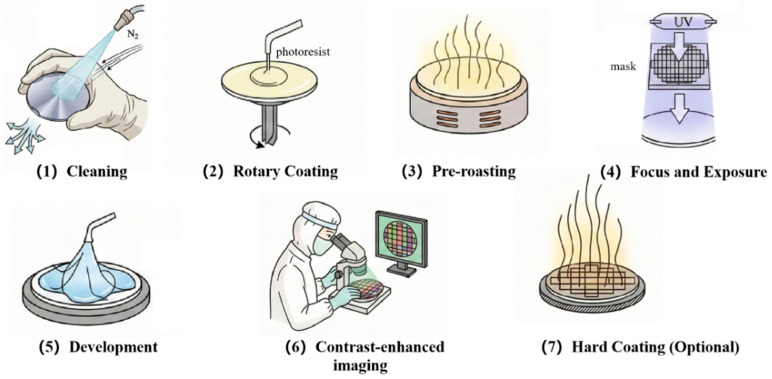
Photolithography process flow.

The PTE detectors are fabricated according to the MEMS microfabrication process flow shown in Figure 2. As can be seen, the fabrication process requires two photolithography steps. Photolithography transfers the design pattern onto a silicon wafer sputtered with Ag_2_Se, preparing it for subsequent physical vapor deposition and peeling processes. The main steps of photolithography are shown in Figure 3.

The following are the detailed steps for the photolithography process:

(1) Cleaning: Pass the Ag_2_Se-sputtered silicon wafers sequentially through acetone to remove organic contaminants and ethanol to replace residual solvents, then rinse with deionized water, and finally dry the wafers with nitrogen.

(2) Spin Coating: Apply photoresist to the Ag_2_Se-sputtered silicon wafers. Spin coating is a commonly used method that utilizes high-speed rotation to ensure the photoresist is evenly distributed across the wafer surface. The type of photoresist and spin coating speed are selected based on specific process requirements. In this step, positive photoresist is used to achieve high-resolution patterns. For thin-film processes, RDP-2100P photoresist is selected for spin coating; ensure the photoresist surface is smooth and free of bubbles. The parameters are set as follows: low speed (200 rpm) for 3 s, 500 rpm for 5 s, high speed (3000 rpm) for 60 s, and 4000 rpm for 5 s, with a film thickness of 7 μm. The uniformity of the photoresist coating is critical to the quality of the subsequent pattern, so precise control of the spin-coating parameters is essential.

(3) Pre-bake: The wafer coated with photoresist requires a pre-bake, which is typically performed on a hot plate set to 100 °C. For RDP-2100P photoresist, the pre-bake time is 1 min. The pre-bake process must be precisely controlled to enhance the effectiveness of subsequent exposure and development.

(4) Alignment and Exposure: Alignment and exposure are performed after pre-baking. Alignment refers to precisely aligning the pattern on the mask with the pattern on the wafer; this is key to ensuring the success of the multilayer lithography process. Exposure involves using ultraviolet light to irradiate the photoresist through the mask, causing a chemical reaction in the illuminated areas of the photoresist. In this process, for RDP-2100P photoresist, the exposure dose is set to 120 mJ/cm^2^.

(5) Development: Development involves removing the photoresist from specific areas after exposure, thereby revealing the desired pattern. A tetramethylammonium hydroxide solution is typically used as the developer. For the spin-coating parameters of the RDP-2100P photoresist, the developer-to-water ratio is 1:8, and the development time is 60 s. The development process must be conducted under strictly controlled conditions to ensure the accuracy and integrity of the pattern.

(6) Development Inspection: An inspection must be performed after development to ensure the accuracy and quality of the pattern. Inspecting alignment accuracy, pattern dimensions, resolution, and edge sharpness using an optical microscope is an essential step. If any defects are found, such as pattern distortion or misalignment, rework and adjustments are required to ensure process precision and product quality.

(7) Post-development baking: Post-development baking further cures the photoresist through heating, enhancing its adhesion and chemical resistance. This step is typically performed on a hot plate at 120 °C for 15 min. Post-development baking not only reduces photoresist diffusion during development but also improves pattern resolution and stability, ensuring the integrity and accuracy of the pattern in subsequent processes.

To meet the requirements for subsequent wire bonding and performance testing, individual devices must be separated from the wafer after the entire fabrication process is complete; this is achieved through a dicing process. For this dicing operation, a DAD322 dicing machine manufactured by DISCO of Japan was selected, utilizing a soft-blade cutting method to separate the devices. During the dicing process, the soft blade moves strictly along a preset path to precisely cut the wafer, ultimately yielding individual, independent devices.

Compared to traditional cutting methods, soft-cutting offers significant advantages. Its excellent cooling effect effectively reduces the instantaneous heat generated during the cutting process, preventing thermal damage to the device caused by high temperatures. Furthermore, this cutting method imposes minimal mechanical stress on the material, minimizing the formation of microcracks and various defects to the greatest extent possible, thereby ensuring the structural integrity of the device. Test results of the dicing process indicate that the dimensions of the individual separated devices match the layout design specifications. The devices exhibit a lateral structure, with the left side appearing darker due to the presence of a structural array, meeting the requirements for subsequent processing and testing.

### 2.2. Characterization and Measurement

The surface topography of Ag_2_Se-based detectors was investigated using a field-emission scanning electron microscope (FESEM; JSM-7800F, JEOL Ltd., Tokyo, Japan). All output performance tests of the PTE were performed in a dark box. A digital source meter (Keithley 2450, Tektronix, Beaverton, OR, USA) was used to measure the current–voltage (I-V) curve under continuous laser, and a pulsed laser was generated using an electronic shutter (DHC GCI-73M, Daheng Optics, Beijing, China) to collect the voltage–time (V-T) curve. Four lasers with different wavelengths (405, 650, 808, and 950 nm) were used during the measurements. And a laser power meter (MC-PM100D, Beijing Merry Change Technology Co., Ltd., Beijing, China) was used to determine the laser power. The laser power was adjusted by changing the input voltage.

## 3. Results and Discussion

**Figure 4:**
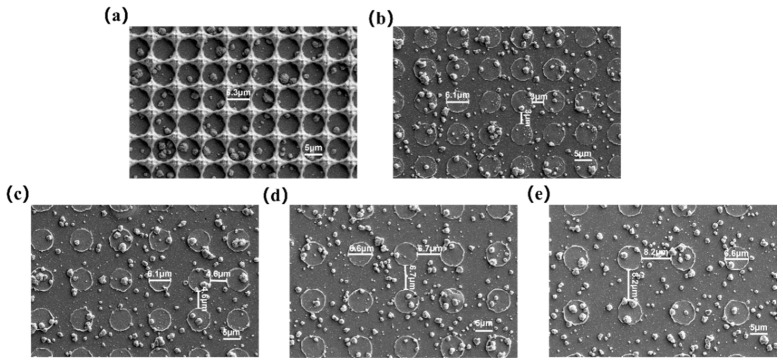
SEM image of the surface functional layer structure: (**a**) structure without prominent features, (**b**) 3 μm spacing, (**c**) 4.6 μm spacing, (**d**) 6.7 μm spacing, (**e**) 8.2 μm spacing.

Figure 4 shows SEM images of surface functional layer structures with different dimensions. The patterning of these structures was achieved through photolithography, peeling, and magnetron sputtering processes. Figure 4a illustrates a case of failed peeling, where the structure failed to form a distinct raised morphology. Figure 4b–e are SEM images of structures with different dimensions. A distinct structural array is visible, with spacing of 3 μm, 4.6 μm, 6.7 μm, and 8.2 μm, respectively, corresponding to diameters of 6.1 μm, 6.1 μm, 6.6 μm, and 6.6 μm, respectively.

**Figure 5:**
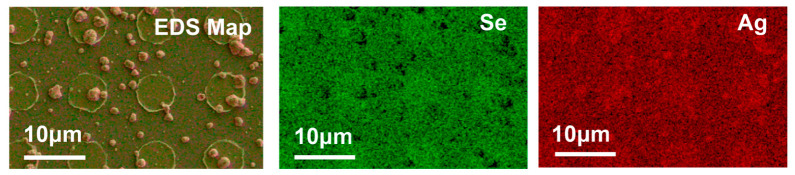
EDS characterization of the surface structural layers of Ag_2_Se.

Energy-dispersive X-ray spectroscopy (EDS) was used to perform a qualitative analysis of its elemental composition. As shown in Figure 5, Ag and Se can be clearly detected within the test area of the microstructural layer on the Ag_2_Se surface; these two elements are the primary constituents of this microstructural layer.

**Figure 6:**
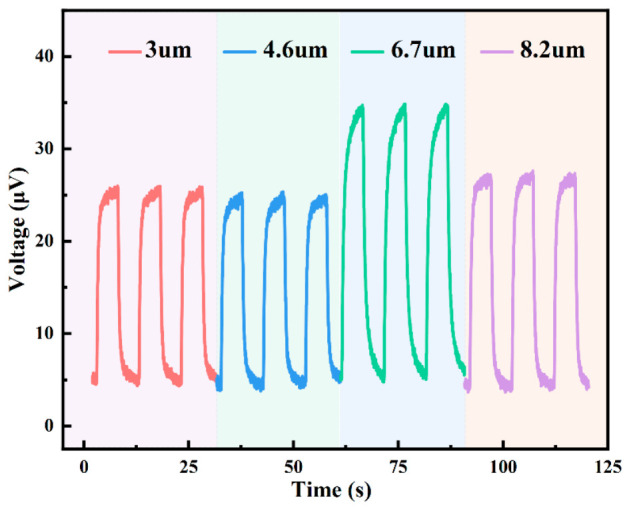
Output voltage under identical illumination conditions at different spacing.

Using a near-infrared laser with a wavelength of 950 nm as the light source, the V-T curves of devices with different spacing structures were measured under constant optical power density conditions. All curves exhibited signals synchronized with the optical on/off switching, confirming the universality of the PTE-electric response. The results in Figure 6 show that the device output voltage reaches its maximum when the structural spacing is 6.7 μm.

**Figure 7:**
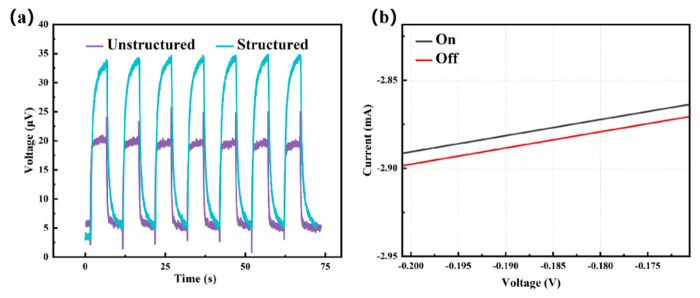
Performance comparison between Device 1 and non-structured devices and I-V characteristics curves: (**a**) performance comparison, (**b**) I-V curves.

Compared to unstructured devices, the performance improvement of the device with a structural pitch of 6.7 μm is shown in Figure 7a, which validates the practical effectiveness of micro–nano structured design. To comprehensively evaluate Ag_2_Se-based detectors with a structured surface functional layer, all subsequent analyses focus on the device with a structural pitch of 6.7 μm (referred to as Device 1). Figure 7b shows the I-V characteristic curves of Device 1. The voltage was scanned under dark conditions and under irradiation with a laser power density of 120 mW/cm^2^ at a wavelength of 950 nm. Both curves exhibit good linearity and are parallel to each other within the measurement range. The linear I-V relationship confirms that an ohmic contact has formed between the metal electrodes and the Ag_2_Se thin film. The parallel shift in the curves before and after illumination is a typical feature of the PTE effect. It indicates that illumination did not alter the device’s internal resistance but instead generated a constant open-circuit voltage. This rules out the possibility of photovoltaic or photoconductive effects as the primary response mechanisms, as the latter typically result in changes to the curve’s slope or shape.

**Figure 8:**
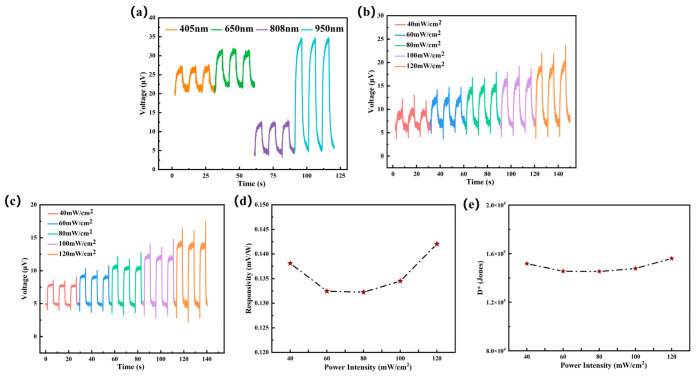
Photothermoelectric response characteristics of the Ag_2_Se-based detectors. (**a**) Output voltage response of the structured photothermoelectric detector under different incident wavelengths. (**b**) Output voltage response of the structured photothermoelectric detector under 950 nm laser irradiation at different power densities. (**c**) Output voltage response of the unstructured photothermoelectric detector under 950 nm laser irradiation at different power densities. (**d**) Power-density-dependent voltage responsivity of the structured photothermoelectric detector. (**e**) Power-density-dependent specific detectivity of the structured photothermoelectric detector.

To further evaluate the overall performance of devices with surface functional layer structures and investigate their operating mechanisms, the output voltage was systematically measured at discrete laser wavelengths of 405, 650, 808, and 950 nm. Further analysis of the device’s PTE performance as a function of incident wavelength is shown in Figure 8a. The device exhibited detectable PTE responses across the entire measurement wavelength range, confirming its detection capability. At 808 and 950 nm, the device exhibited a small dark voltage, whereas at 405 and 650 nm, the dark voltage was relatively large. The maximum photoresponse was achieved under 950 nm laser irradiation, with an output voltage increase of 29.7 μV, and the response curve exhibited a distinct peak. Although there is still room for improvement in the PTE performance of the current device, it has demonstrated detection capabilities across different spectral ranges, validating the effectiveness and potential of micro–nanostructures in regulating the PTE performance of PTE detectors. At a laser wavelength of 950 nm, the photoresponse output voltage of the corresponding device was measured by varying the incident light power density. To further compare the performance of the structured and unstructured Ag_2_Se photothermoelectric detectors, the output voltage responses of both devices were measured under 950 nm laser irradiation at different power densities. As shown in Figure 8b,c, with increasing laser power density, both devices exhibit obvious photothermoelectric responses, confirming their power-dependent detection capability. Compared with the unstructured device, the structured detector shows a higher output voltage under the same illumination conditions. This improvement can be attributed to the surface microstructure, which increases the effective light absorption and local photothermal conversion, thereby enhancing the temperature gradient and photothermoelectric voltage output. As shown in Figure 8d, the sensitivity value exhibits a non-monotonic trend with increasing incident light power density rather than remaining constant. At a laser power density of 120 mW/cm^2^, the maximum sensitivity is approximately 0.14 mV/W. This is because at lower laser power densities, the amplitude of the PTE signal generated by the device is small and may be on the same order of magnitude as the background noise of the measurement system, leading to significant fluctuations in the responsivity value and making it difficult to accurately reflect its inherent trend. As the laser power density increases, the PTE conversion efficiency improves, and the responsivity peaks at 120 mW/cm^2^. The thermal-noise-limited specific detectivity of the structured Ag_2_Se photothermoelectric detector was further estimated. The thermal noise voltage spectral density was calculated using ($v_{n}=\sqrt{4k_{B}RT}$) with a measured device resistance of 50 Ω at room temperature. The noise-equivalent power and specific detectivity were estimated using ($NEP=v_{n}/R_{v}$) and ($D^{*}=1/NEP$), respectively. As shown in Figure 8e, based on the voltage responsivity measured at 950 nm, the calculated (D^*^) ranges from (1.45 × 10^5^) to (1.56 × 10^5^) Jones under laser power densities of 40–120 mW cm^−2^, with the maximum value obtained at 120 mW cm^−2^. This value represents a thermal-noise-limited estimation, and full noise spectral density measurements will be performed in future work. The relatively high incident power density used in this work is mainly associated with the small voltage output of the present prototype device, thermal losses in the lateral device geometry, and the limited readout sensitivity of the current testing setup. Therefore, the present Ag_2_Se-based photothermoelectric detector should be considered as an initial demonstration of surface-structure-induced performance modulation rather than a device with an already-optimized detection limit. Future work will focus on improving light absorption, reducing thermal dissipation, optimizing the device geometry and readout configuration, lowering the measurable power threshold, and systematically comparing structured and unstructured devices over a broader power-density range.

**Figure 9:**
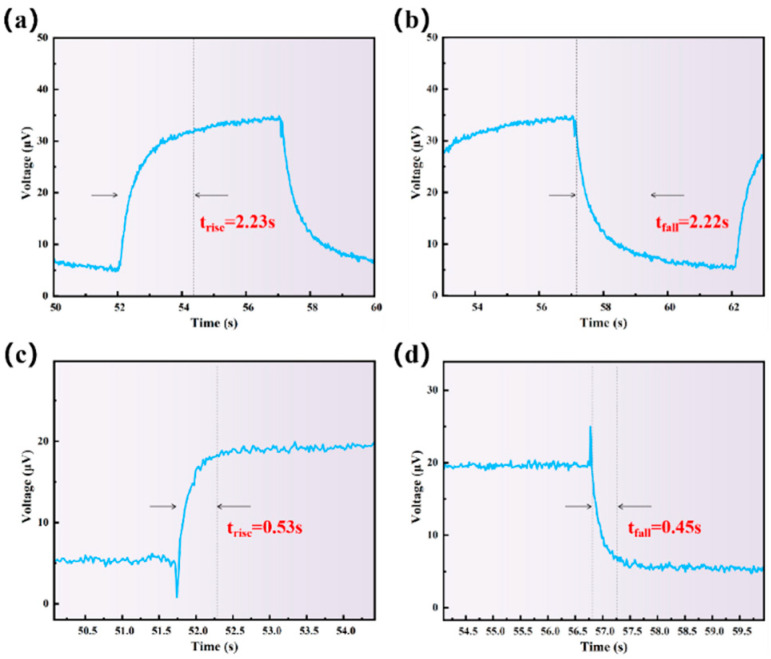
Photovoltaic response time characteristics: (**a**) photovoltaic rise curve of Device 1, (**b**) photovoltaic fall curve of Device 1, (**c**) photovoltaic rise curve of the unstructured device, (**d**) photovoltaic fall curve of the unstructured device.

The study systematically compared the dynamic response behavior of Device 1 with that of an unstructured device under laser irradiation to investigate the mechanism by which the patterning of the surface functional layer affects the device’s transient performance. In the tests, a 950 nm laser source was used to capture the complete output voltage trajectory of the device before and after laser switching and to extract key dynamic parameters including rise time and fall time. Rise time and fall time are defined as the time intervals during which the photovoltage rises from 10% to 90% and decays from 90% to 10%, respectively. The analysis results reveal a key physical principle: while micro–nanostructures effectively enhance the device’s steady-state PTE output voltage, they inevitably lead to a slowing of its dynamic response speed. Figure 9a,b show the rise and fall curves of the light-response output voltage at 0 V bias; Device 1 exhibits rise and fall times of 2.23 s and 2.22 s, respectively. In contrast, Figure 9c,d demonstrate the dynamic characteristics of the unstructured device, which has response rise and fall times of only 0.53 s and 0.45 s, respectively, indicating a significantly faster response speed. This significant difference clearly shows that the performance improvement from micro–nanostructures comes at the cost of response speed. It reveals an inherent trade-off between two core performance indicators of photothermal devices: response sensitivity and response speed. This trade-off is governed by fundamental physical principles. As summarized in Table 1, the present structured Ag_2_Se-based photothermoelectric detector exhibits a detectable spectral response from 405 to 950 nm, with a voltage sensitivity of approximately 0.14 mV W^−1^ and rise/fall times of 2.23/2.22 s. Although its sensitivity is lower than that of some reported photothermoelectric detectors, its response speed is relatively faster than most compared devices. This result indicates that the lithography-defined surface structure can effectively modulate the PTE output while maintaining an acceptable dynamic response. Further optimization of light absorption, thermal management, and device geometry is expected to improve the sensitivity and overall detection performance.

## 4. Conclusions

In this work, Ag_2_Se-based photothermoelectric detectors with different surface structural pitches were fabricated using photolithography, lift-off, and magnetron sputtering processes. The influence of surface microstructure on the PTE response performance was systematically investigated. The results show that the device with a structural pitch of 6.7 μm exhibits the best overall performance and achieves a detectable photoresponse over the measured spectral range of 405–950 nm. Under 950 nm laser irradiation, the structured detector delivers a higher output voltage than the unstructured detector at different power densities, confirming that the surface microstructure can enhance light absorption, local photothermal conversion, and temperature-gradient-induced thermoelectric output. At a laser power density of 120 mW cm^−2^, the structured device achieves a maximum voltage sensitivity of approximately 0.14 mV W^−1^ and a thermal-noise-limited specific detectivity of 1.56 × 10^5^ Jones. However, the dynamic response comparison shows that the structured device has longer rise and fall times than the unstructured device, indicating a trade-off between enhanced steady-state output and response speed. This slower response is mainly attributed to the increased thermal diffusion path and thermal relaxation process introduced by the surface microstructure. Overall, this work demonstrates that surface functional layer structuring is an effective strategy for regulating the performance of Ag_2_Se-based PTE detectors. Future work will focus on improving light absorption, reducing thermal dissipation, optimizing device geometry and readout configuration, lowering the measurable power threshold, and extending the detectable spectral range toward longer wavelengths.

## Figures and Tables

**Table 1:** Performance comparison of the present Ag_2_Se-based photothermoelectric detector with recently reported photothermoelectric detectors.

No.	Material	Spectral Range	Responsivity	Response Time	Ref.
1	Ag_2_Se	405–950 nm	0.14 mV/W	2.23/2.22 s	This work
2	Ag_2_Se	400–950	/	~60 s	[21]
3	AZO	1000–1500 nm	1.5 mV/W	~150 s	[22]
4	Doped polyaniline/graphene	/	2.5 V/W	~10 s	[23]
5	Graphene/PEDOT	/	2.27 mV/W	6 s	[24]

## Data Availability

Data will be made available on request.
